# Drug-resistant tuberculosis and advances in the treatment of childhood tuberculosis

**DOI:** 10.1186/s41479-016-0019-5

**Published:** 2016-11-24

**Authors:** James A. Seddon, H. Simon Schaaf

**Affiliations:** 1 Centre for International Child Health, Department of Paediatrics, Imperial College London, London, UK; 2 Department of Paediatrics and Child Health, Desmond Tutu TB Centre, Faculty of Medicine and Health Sciences, Stellenbosch University, Cape Town, South Africa

**Keywords:** Tuberculosis, Children, Resistant, Treatment, Disease, Infection

## Abstract

Over the last 10 years, interest in pediatric tuberculosis (TB) has increased dramatically, together with increased funding and research. We have a better understanding of the burden of childhood TB as well as a better idea of how to diagnose it. Our appreciation of pathophysiology is improved and with it investigators are beginning to consider pediatric TB as a heterogeneous entity, with different types and severity of disease being treated in different ways. There have been advances in how to treat both TB infection and TB disease caused by both drug-susceptible as well as drug-resistant organisms. Two completely novel drugs, bedaquiline and delamanid, have been developed, in addition to the use of older drugs that have been re-purposed. New regimens are being evaluated that have the potential to shorten treatment. Many of these drugs and regimens have first been investigated in adults with children an afterthought, but increasingly children are being considered at the outset and, in some instances studies are only conducted in children where pediatric-specific issues exist.

## Background

### How do children get tuberculosis?

If a child is exposed to an individual, usually an adult, with infectious pulmonary tuberculosis (TB) disease they are at risk of inhaling aerosolised *Mycobacterium tuberculosis* and becoming infected. Whether they become infected or not following exposure will depend on the integrity of their mucosal defences, their innate immune system, the virulence of the mycobacterium and the infective dose. Once infection has occurred the adaptive immune system recognises the bacilli and may clear the organism, become overrun by it or reach an equilibrium in which the immune system fails to eradicate the mycobacteria but prevents them from proliferating. This final situation is termed TB infection. In the future, the bacilli may overcome the immune system and progress to TB disease [1–3].

Other than occasionally having brief, viral-like symptoms, children with TB infection usually have no clinical symptoms or signs, and radiology shows no evidence of TB disease. TB infection is detected through a positive tuberculin skin test (TST) or interferon-gamma release assay (IGRA). The risk of progressing from infection to disease is governed by a number of factors but age and immune status are central. From studies that examined the natural history of TB, conducted prior to the chemotherapy era, we know that infected infants have a 50% risk of progression to disease, with the risk decreasing with age through childhood but increasing again as children enter adolescence [4, 5]. HIV-positive adults not on antiretroviral therapy have a 7–10% risk of developing TB each year following TB infection; [6, 7] the risk is likely to be similar for children. Children with malnutrition or other forms of immune deficiency have also been shown to be more vulnerable [8]. If children are identified at the point that they have TB infection, the risk of progression to disease can be markedly reduced by giving preventive therapy.

**Fig. 1:**
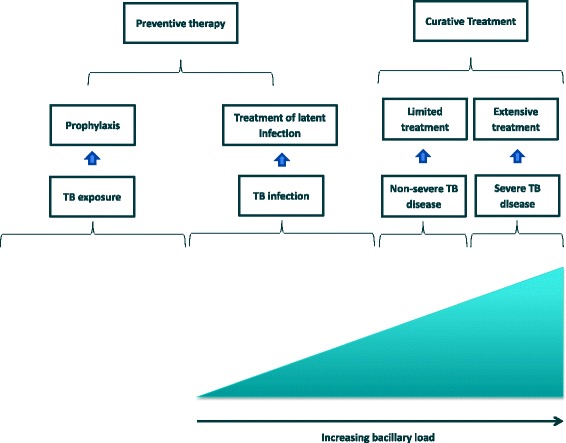
The continuum of tuberculosis exposure, infection, non-severe and severe disease in children and possible treatment implications

Children with TB disease have a wide range of clinical presentations. The most common presentation in young children is intra- or extra-thoracic lymph node disease. However, young children (<3 years) are also more likely than older children or adults to develop the most severe forms of disseminated TB, such as TB meningitis or miliary TB. As children get older (starting from about 8 years of age) they are more likely to develop adult-type disease, including cavitary pathology. Due to this variety of clinical forms, investigators are increasingly exploring whether it is possible to divide children into those with severe disease and non-severe disease, using consistent definitions, with the possibility that those with non-severe pathology might be treated with fewer drugs and for shorter durations (Fig. 1) [9].

### How many children in the world have TB?

This topic is covered in detail in the article by Jenkins in this series [10]. Multidrug-resistant (MDR)-TB is defined as disease caused by *M. tuberculosis* resistant to rifampicin and isoniazid, whereas extensively drug-resistant (XDR)-TB is defined as disease caused by MDR organisms with additional resistance to a fluoroquinolone and a second-line injectable medication. The World Health Organization (WHO) estimates that 1 million children developed TB in 2014 [11]. Only 358, 521 children were diagnosed, treated and reported to the WHO that year, suggesting that about two thirds of the children that develop TB each year remain undiagnosed, untreated or were not reported. Investigators have estimated that about 30,000 children develop MDR-TB each year [10, 12, 13]. Given that only 1000 children have been described in the entire medical literature as having been treated for MDR-TB at any point [14], under-diagnosis and under-treatment is likely to be even worse for MDR-TB.

### Diagnosing TB infection and TB disease

Both TB infection and TB disease can be challenging to diagnose with certainty in children [15]. The TST and IGRA are associated with impaired sensitivity and specificity in children; [16–19] children can therefore be assumed to have TB infection if they have been heavily exposed to an infectious case of TB. If they are at high risk of disease progression (< 5 years of age or HIV-infected) and they have been exposed to a case of drug-susceptible TB, then WHO recommends that they be given preventive treatment without the need for TST or IGRA testing [20]. In most contexts only a small proportion of the children (often fewer than 30%) that are treated for TB disease have a bacteriologically confirmed diagnosis [21]. Treated cases are therefore confirmed or presumed. For research purposes, investigators have tried to quantify the confidence that is given to the diagnosis of presumed TB and comprehensive definitions have been developed through consensus to describe confirmed, probable and possible TB for both drug-susceptible (DS) [22] and drug-resistant (DR) TB disease [23]. For children presumed to have DR-TB, multiple microbiological samples should be taken, ideally before treatment. Once samples are taken, however, the child should be treated with a regimen designed on the assumption that they have the same drug susceptibility test (DST) pattern as the identified source case [24, 25].

## Treating drug-susceptible tuberculosis infection

### What is the recommended treatment of drug-susceptible TB infection (LTBI) in children?

Isoniazid given for 6 or 9 months has been shown to be very effective in preventing the progression from TB infection to disease [26] and a number of studies demonstrate that 3 months of isoniazid and rifampicin is also an effective regimen [27]. Rifampicin alone is likely to be effective if given for 3 or 4 months [28]. However, providing daily therapy to a child who is clinically well can be challenging for many parents; adherence is frequently poor, particularly in high burden settings [29, 30].

### Are there any alternatives?

In 2011, the results of a large trial were published which had evaluated once weekly rifapentine and high-dose isoniazid for 3 months (12 dosing episodes) against 9 months of daily isoniazid [31]. This demonstrated that the shorter, once-weekly regimen was as effective in preventing TB disease as a 9-month daily isoniazid regimen and was also associated with better adherence. Although the study did include children above the age of 2 years, the investigators did not feel that there were sufficient children in the trial to be confident of the adverse events profile in pediatric populations. To that end, the study continued to recruit children for a further 2 years until more than 1000 children had been enrolled [32]. This found the 3-month regimen to be associated with higher completion rates and limited toxicity. Detailed pharmacokinetic studies and extensive modelling provide good evidence for the best dosage to give children when using either whole tablets or crushed tablets [33]. This regimen should still be evaluated in the most vulnerable age group of less than 2 years.

## Treating drug-susceptible tuberculosis disease

### What is the recommended treatment of drug-susceptible TB disease in children?

The WHO recommends that children with pulmonary DS-TB are treated with 2 months of rifampicin, isoniazid and pyrazinamide followed by 4 months of rifampicin and isoniazid. They advise that ethambutol should be added for the first 2 months in children with extensive disease or where rates of HIV infection and/or isoniazid resistance are high, irrespective of the child’s age [20]. This regimen is effective and is associated with few adverse events; [34] optic neuritis is an extremely rare adverse effect at the dosages advised [35]. Due to emerging pharmacokinetic evidence, the recommended dosages of these first-line anti-TB medications were revised in 2010 as children metabolise the drugs more rapidly than adults resulting in a lower serum concentration following the same mg/kg dosage [36]. It is only using the revised dosages that young children achieve the target serum concentrations that have been shown to be associated with efficacy in adult studies [37]. Following the 2010 revision of pediatric TB dosing recommendations, the ratio of individual medications included in the fixed dose combination (FDC) tablets similarly required updating. A new appropriately dosed, scored, dispersible and palatable pediatric FDC tablet was launched in December 2015; these tablets are expected to be available for use by the end of 2016 [38].

### Is it possible to shorten TB treatment?

Six months is a long time to treat a child and a number of adult studies have recently been completed that aimed to shorten treatment to 4 months using alternative regimens. In the RIFAQUIN trial adults were randomised to one of three regimens: (i) the traditional 6-month WHO-recommended regimen; (ii) 2 months of daily ethambutol, moxifloxacin, rifampicin and pyrazinamide followed by 2 months of twice weekly moxifloxacin and rifapentine; and (iii) 2 months of daily ethambutol, moxifloxacin, rifampicin and pyrazinamide followed by 4 months of once weekly moxifloxacin and rifapentine [39]. Although the 4-month regimen was inferior to the standard course of treatment (more patients relapsed), the alternative 6-month regimen, in which patients only had to take treatment once a week in the continuation phase, was non-inferior. This raises the exciting prospect of once weekly treatment for children in the continuation phase of treatment. The OFLOTUB trial compared the standard 6-month regimen with a new experimental regimen in adults, in this case gatifloxacin, rifampicin and isoniazid for 4 months with additional pyrazinamide for the first 2 months [40]. As with the RIFAQUIN trial, the shortened regimen was found to be inferior with more unfavourable outcomes (death, treatment failure, recurrence) in the shorter treatment group. However, there was great variation by country and also by HIV status and body mass index (outcomes were similar between the two treatments for malnourished patients and those with HIV). This suggests that there may be a role for shortened treatment in some patient populations or it might work in certain health systems. The final adult study, the REMox trial, compared the WHO adult first-line regimen to two experimental arms: (i) 4 months of moxifloxacin, isoniazid and rifampicin with additional pyrazinamide for the first 2 months; and (ii) 4 months of moxifloxacin and rifampicin with ethambutol and pyrazinamide for the first 2 months. More rapid culture-conversion was seen in the moxifloxacin-containing arms but the shortened regimens were inferior to the WHO regimen [41].

A pediatric trial, SHINE, is due to soon start at a number of sites in Africa, and also in India, that will evaluate whether children with non-severe disease can be treated successfully with only 4 months of treatment [42]. If more effective contact tracing occurs following the diagnosis of TB in adults, it is expected that more children with TB will be detected at an earlier stage in their disease process. If these children can be safely treated with shorter treatment regimens, better adherence and cheaper treatments would be expected.

### What is the best treatment for TB meningitis?

The WHO suggests that children with TB meningitis (TBM) should be treated for 2 months with isoniazid, rifampicin, pyrazinamide and ethambutol followed by 10 months with isoniazid and rifampicin at the standard dosages [20]. There are concerns that this regimen may not be ideal. Isoniazid and pyrazinamide penetrate well into the cerebrospinal fluid (CSF), rifampicin penetrates moderately when there is meningeal inflammation and poorly after this has subsided, with ethambutol having almost no penetration [43–45]. Therefore, during the first 2 months of treatment two drugs are being given with good CSF penetration and for the subsequent 10 months effectively only one drug is being given. In areas of increased rates of isoniazid resistance, many children are left without any effective treatment after the first 2 months. Further, the dosages recommended for treatment do not fully consider the penetration into the CSF and it is expected that higher dosages are required to achieve adequate CSF concentrations. Outcomes for children with TBM are very poor [46]. One group in Cape Town, South Africa, have been treating TBM in children with a short, intensive regimen for a number of years [47–49]. This consists of high-dose isoniazid (15-20 mg/kg), rifampicin (20 mg/kg), pyrazinamide (40 mg/kg) and ethionamide (20 mg/kg) for 6 months. Outcomes are reasonable and the regimen is well tolerated. Although an exciting trial in adults with TBM in Indonesia showed that high dosages of rifampicin (given intravenously) combined with moxifloxacin improved outcome [50], a further study in Vietnam failed to demonstrate a protective effect of higher dose rifampicin and the addition of levofloxacin. A pediatric trial, TBM-KIDS, has started in Malawi and India and aims to evaluate the pharmacokinetics, safety and efficacy of levofloxacin and high-dose rifampicin in TBM [51].

The role of immune modulators in pediatric TBM is still unclear. A number of trials have demonstrated that the use of steroids offers a modest benefit on death and severe disability [52]. However, this may be restricted to only those with certain host genotypes [53] and the dosage to give children remains unclear [54]. A trial of high-dose thalidomide as an immune modulator in TBM was stopped early due to worse outcomes in the intervention group [55]. However, thalidomide at a lower dose has since been used successfully in the treatment of optochiasmatic arachnoiditis and tuberculomas/pseudoabscesses in children [56, 57]. The effect of aspirin is unclear. In one pediatric trial aspirin demonstrated a benefit [58], whereas in another it did not [59].

## Treating drug-resistant tuberculosis infection

### How does drug-resistant TB develop?

Drug resistance can be acquired through sequential, selective pressure in the face of inadequate therapy. Here, spontaneously occurring mutants are favoured that provide resistance against individual drugs. This process usually takes place in the presence of a high bacillary load, where previously drug-susceptible organisms develop resistance within one human host. Alternatively, resistance can be transmitted where mycobacteria, already resistant, are transmitted to a new host. Additionally, a combination of the two can occur when one individual receives a mycobacterium already resistant to one or more medications and then in the face of inadequate treatment develops resistance to further antibiotics (resistance amplification). Children usually have transmitted resistance, as disease is normally paucibacillary, making acquired resistance less likely.

### How should we investigate a child who has been exposed to a drug-resistant TB source case?

If a child has been exposed to an infectious source case with DR-TB they should be assessed for evidence of TB disease. This would include a comprehensive symptom screen, clinical examination and, where available, chest radiography. Any concerns that the child has TB disease should necessitate further investigation. If the child is symptom-free, growing well, with no concerning clinical signs, they should be evaluated for risk of infection. Where available, TST and/or IGRA could be employed to evaluate the risk of infection but if they are unavailable an assessment can be made on the basis of exposure.

### How should we treat a healthy child who has been exposed to a drug-resistant TB source case?

Children exposed to either rifampicin mono-resistant TB or isoniazid mono-resistant TB can usually be given either isoniazid or rifampicin alone, respectively. The correct management of children exposed to MDR-TB is unclear [60], with a limited evidence base to support policy [61, 62]. Using isoniazid and/or rifampicin (the two drugs for which there is a strong evidence base for preventive therapy) is unlikely to be effective [63] as the organism is, by definition, resistant to these drugs. International guidelines are highly variable [64]. The British National Institute for Health and Care Excellence advises follow up with no medical treatment [65], as does the WHO [66]. The US Centers for Disease Control and Prevention (CDC), the American Thoracic Society and the Infectious Diseases Society of America advise giving two drugs to which the source case’s strain is susceptible [67]. The European Centre for Disease Prevention and Control suggest that either treatment or close follow up are legitimate options [68].

Only a few studies have assessed preventive therapy in MDR-TB child contacts. In Israel, 476 adult and child contacts of 78 pulmonary MDR-TB patients were evaluated. Twelve were given a tailored preventive therapy regimen, 71 were given isoniazid, six were given other treatments and 387 were not given any treatment. No contacts developed TB [69]. In Cape Town, from 1994 to 2000, 105 child contacts of 73 MDR-TB source cases were identified and followed up. Two (5%) of the 41 children who received tailored preventive therapy developed TB as opposed to 13 (20%) of the 64 children who were not given any [70]. In a retrospective study in Brazil, 218 contacts of 64 MDR-TB source cases were given isoniazid, while the remainder were observed without treatment. The rate of TB was similar in the group who were given isoniazid (1.2 per 1000-person-months of contact) compared to those who were not (1.7 per 1000-person-months of contact; *p* = 0.47). In two outbreaks in Chuuk, Federated States of Micronesia, five MDR-TB source cases were identified. Of 232 contacts identified, 119 were offered preventive therapy, of which 104 initiated a fluoroquinolone-based regimen. None of those who started preventive therapy developed TB disease, whereas three of the 15 who did not take treatment did [68, 71]. A prospective study from Cape Town recruited 186 children during 2010 and 2011 who had been exposed to adult source cases with MDR-TB. All were offered three-drug preventive therapy with ofloxacin, ethambutol and high dose isoniazid. Six children developed TB and one infant died. Factors associated with poor outcome were: age less than 12 months, HIV infection and poor adherence [72]. Although a clinical trial is urgently needed to assess how to best manage children exposed to MDR-TB, these studies together suggest that providing preventive therapy may be effective in stopping the transition from infection to disease. Three randomised trials are planned. VQUIN are recruiting adult contacts of MDR-TB in Vietnam and randomising them to either levofloxacin or placebo. TB-CHAMP will take place in four sites in South Africa and recruit children under 5 years of age following MDR-TB household exposure. This trial will also randomise contacts to levofloxacin or placebo. PHOENIx will take place at a number of sites globally and recruit adults and children with all patients randomised to either delamanid or isoniazid. Although the results of these trials are eagerly awaited, an expert group, convened in Dubai in 2015, concluded that there is currently enough observational evidence to treat high risk contacts with a fluoroquinolone-based regimen [73].

### How should we follow up these children?

As 90% of children who develop TB disease do so within 12 months and as almost all do so within 2 years [74], follow up for at least 12 months is advisable whether preventive therapy is given or not. The WHO and several other guidelines recommend 2 years of follow up. Clinical follow-up is likely sufficient but where resources permit, chest radiology at 3–6-month intervals can detect early disease when symptoms may not be obvious.

## Treating drug-resistant tuberculosis disease

### How do you design a regimen for a child in order to treat for drug-resistant TB?

**Fig. 2:**
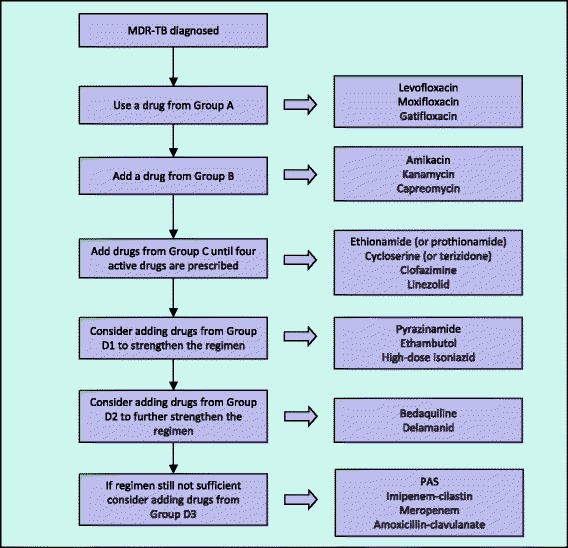
Constructing a regimen for the treatment of a child with multidrug-resistant tuberculosis

In 2016 the WHO updated its recommendations for the management of MDR-TB [75]. It also re-structured the groupings into which the different drugs were placed (Table 1). Drugs are added to the regimen in the following order (as long as the drug is likely to be effective): first a fluoroquinolone is added (WHO Group A), followed by a second-line injectable medication (Group B). Further drugs from Group C are added until four likely effective drugs are present. To strengthen the regimen or to provide additional drugs to make four effective drugs, agents from Group D can be added (Fig. 2).

**Table 1:** New drug groupings published by the World Health Organization in 2016 [75]

	Drug group		Drug name	Abbreviation	Important Adverse Events
A	Fluoroquinolones		Levofloxacin	Lfx	Sleep disturbance, GI disturbance, arthritis, peripheral neuropathy,
			Moxifloxacin	Mfx	As levofloxacin with QTc prolongation
			Gatifloxacin	Gfx	As levofloxacin with QTc prolongation
B	Second-line injectable agents		Amikacin	Am	Ototoxicity, nephrotoxicity
			Kanamycin	Km	As amikacin
			Capreomycin	Cm	As amikacin
C	Other core second-line agents		Ethionamide/Prothionamide	Eto/Pto	Gastrointestinal disturbance, metallic taste, hypothyroidism
			Cycloserine/Terizidone	Cs/Trd	Neurological and psychological effects
			Linezolid	Lzd	Diarrhoea, headache, nausea, myelosuppression, neurotoxicity, optic neuritis, lactic acidosis and pancreatitis
			Clofazimine	Cfz	Skin discoloration, abdominal pain, QTc prolongation
D	Add-on agents	D1	Pyrazinamide	Z	Arthritis/arthralgia, hepatitis
			Ethambutol	E	Optic neuritis
			High-dose isoniazid	hH	Hepatitis, peripheral neuropathy
		D2	Bedaquiline	Bdq	Headache, nausea, liver dysfunction, QTc prolongation
			Delamanid	Dlm	Nausea, vomiting, dizziness, paresthesia, anxiety, QTc prolongation
		D3	*p-aminosalicylic acid*	PAS	GI intolerance, hypothyroidism, hepatitis
			Imipenem-cilastatin	Ipm	GI intolerance, hypersensitivity reactions, seizures, liver and renal dysfunction
			Meropenem	Mpm	As Imipenem
			Amoxicillin-clavulanate	Amx-Clv	As Imipenem

Although for adults it is recommended that the intensive phase (including the injectable agent) should last 8 months and the full duration of therapy should be no less than 20 months, the 2016 WHO guidelines recognise the fact that many children with non-severe disease have been successfully treated with shorter regimens and many with no injectable in the regimen. Given the high rates of irreversible hearing loss, consideration should be given to either omitting the injectable agent or giving it for a shorter period of time (3–4 months) in children with non-severe disease. Total duration of therapy could also be shorter (12–15 months) than for adults.

### How should children being treated for drug-resistant TB be followed up and monitored?

Children should be monitored for three reasons: to determine response to therapy; to identify adverse events early; and to promote adherence. A suggested monitoring schedule, which should be adapted to local conditions and resources, is demonstrated in Table 2.

**Table 2:** Suggested schedule of follow up for children on treatment for multidrug-resistant tuberculosis

All children	Baseline	Month										Ongoing
		1	2	3	4	5	6	9	12	15	18	
HIV status	•											
Toxicity (symptoms, signs)	•	•	•	•	•	•	•	•	•	•	•	•
Height and weight	•	•	•	•	•	•	•	•	•	•	•	•
Audiology^a^	•	•	•	•	•	•	•					
Colour vision testing^b^	•	•	•	•	•	•	•	•	•	•	•	•
Chest radiograph^c^	•			•			•					
TB culture and DST^d^	•	•	•	•	•	•	•					
Creatinine and potassium^a^	•	•	•	•	•	•	•					
TSH, free T_4_^e^	•			•			•	•	•	•	•	•
Haematology (FBC)^f^	•	•	•		•		•	•	•	•	•	•
ECG^g^	•	•	•		•		•	•	•	•	•	•
HIV-positive												
LFTs, Cholesterol	•						•			•		•
CD4 count and viral load	•						•			•		•

*DST* drug susceptibility test, *TSH* thyroid stimulating hormone, *ECG* electrocardiogram, *LFTs* liver function tests

^a^Monthly whilst on an injectable and at 6 months following termination of injectable

^b^If on ethambutol or linezolid

^c^If any pulmonary involvement or at any point if clinically indicated; to be repeated at the end of treatment

^d^Monthly if old enough to expectorate. If unable to expectorate and initially smear or culture positive, monthly until culture-converted then 3 monthly. If initially smear and culture negative, to perform if clinically indicated

^e^if on ethionamide, prothionamide or PAS

^f^if on linezolid or if HIV-positive

^g^if on bedaquiline or delamanid

Response to therapy includes clinical, microbiological and radiological monitoring. Children should be clinically assessed on a regular basis to identify symptoms or signs that might signal response: activity levels, respiratory function and neurological development. Height and weight should be measured monthly and, for children with pulmonary disease, respiratory samples for smear microscopy and culture (not genotypic evaluation during follow-up) should be collected where possible. Children with pulmonary disease should have a chest radiograph at 3 and 6 months and at any time if clinically indicated. It is also useful to have a chest radiograph at the end of therapy to provide a baseline for follow-up.

Children should be assessed clinically for adverse events on a regular basis. Prior to the start of treatment, children should have a baseline assessment of thyroid function, renal function and have audiological and vision examinations. Both ethionamide and para-aminosalicylic acid (PAS) have been shown to cause hypothyroidism [76–81] and the thyroid function should be checked every 2 months. The injectable drugs can cause renal impairment and hearing loss [82–85]. Renal function should be determined every 2 months; hearing evaluation should be done at least every month while on an injectable drug and 6 months after stopping the agent, as hearing loss can continue after discontinuing the drug. The testing of hearing is age-dependent and for those older than five with normal neuro-development, pure tone audiometry (PTA) is the best assessment. Otoacoustic emissions can be used to test the hearing in younger children but visual testing is challenging for this age group. Children being given ethambutol who are able to co-operate with colour vision testing, should be assessed monthly, using an appropriate Ishihara chart. This is usually possible from the age of five. Clinicians should, however, be reassured that the incidence of ocular toxicity is very rare when ethambutol is given at the recommended dosage [86].

### What are the common adverse effects associated with treating children for drug-resistant TB?

Most anti-TB drugs can cause gastrointestinal upset and rash but in most instances, these resolve without treatment and without compromising therapy. Severe cutaneous drug reactions, such as Stevens-Johnson syndrome, necessitate immediate cessation of all drugs until the symptoms have resolved. Gastrointestinal upset is most pronounced with ethionamide and PAS and frequently this can be managed without stopping the drug by dose escalation, by dividing the dose or by anti-emetics in older children/adolescents. If either colour vision or hearing are found to be deteriorating, consideration should be given to stopping the ethambutol (vision) or injectable medication (hearing); if not a failing regimen, substitution with another drug could be considered. If the thyroid stimulating hormone (TSH) is elevated and the free T4 is low then consideration should be given to starting thyroxine substitution. Peripheral neuropathy can be treated by either increasing the dose of pyridoxine or reducing the dose of isoniazid or linezolid. If it persists, the causative drug should be stopped. Determining the cause of neuropsychiatric adverse events can be complicated as many drugs can cause dysfunction. Dose reduction may help, but if symptoms persist the likely drug should be stopped. Joint problems can be caused by pyrazinamide and the fluoroquinolones and management options include reducing or stopping one/both of these drugs. Hepatotoxicity usually starts with new-onset vomiting. Clinical hepatitis (tender liver, visible jaundice) necessitates immediate cessation of all hepatotoxic drugs. These include rifampicin, isoniazid, pyrazinamide, ethionamide, PAS, beta-lactams and macrolides. Treatment should continue with the remaining drugs and consideration given to starting any other available medications that are not hepatotoxic. The hepatotoxic drugs can be re-introduced if felt to be necessary, one-by-one every 2 days, while checking the liver enzymes to identify the possible causative drug(s).

### How successful is treatment for drug-resistant TB in children?

A systematic review and meta-analysis, published in 2012, identified only eight studies reporting the treatment of MDR-TB in children; 315 children were included in the meta-analysis [87]. Successful outcomes were seen in 82% of children, as compared to 62% in adults [88, 89]. It is difficult to draw too many firm conclusions from such small numbers but it does appear that if children are identified, diagnosed and treated with appropriate therapy, outcomes are very good. However, these individualised approaches require high levels of expertise from the clinicians who manage these children, the treatment is long (up to 18 months and longer) and is associated with significant adverse events.

Since this systematic review there have been a large number of publications that have described the treatment of MDR-TB in children [90–108]. In one study from Cape Town, Western Cape children were classified as having had severe or non-severe disease [108]. The children with non-severe disease were younger, better nourished, less likely to have HIV infection, were less likely to have confirmed disease and less likely to have sputum smear-positive TB. They were more commonly treated as outpatients, less likely to receive an injectable medication and were given shorter total durations of medication (median 12 months vs. 18 months in the severe cases). A study from four provinces in South Africa (outside the Western Cape) collected routine data on the treatment of more than 600 children with MDR-TB. Although mortality was slightly higher than in other studies at 20%, these children were often treated outside of specialist centres. In preparation for the revision to the WHO DR-TB guidelines an individual patient systematic review and meta-analysis was commissioned to evaluate the treatment of children with MDR-TB. More than a thousand children were included and treatment outcomes were successful in 77% of cases [14].

In addition to these studies, there have been a number of pharmacokinetic investigations of second-line anti-TB drugs in children [109–111] and novel delivery systems have been designed [112]. A consensus statement has been developed suggesting definitions that could be used in pediatric MDR-TB research [23] and a number of guidelines have been published [113–116], as well as a practical field guide [117].

### Are there any new drugs to treat children for drug-resistant TB?

A couple of antibiotics traditionally used for the treatment of other infections are now more commonly used [118–122] and have been promoted in the WHO drug grouping. Linezolid was shown to be highly effective in adult patients with XDR-TB who were failing therapy [123]. Almost all the adults developed adverse effects, some severe, necessitating cessation of therapy. A systematic review demonstrated that linezolid could be an effective component of DR-TB treatment regimens but is associated with significant adverse events [124]. Linezolid in children seems as effective as in adults, but with fewer adverse events [95, 125–127]. Clofazimine, traditionally an anti-leprosy drug, has also gained a great deal of interest recently mainly due to its central role in the Bangladesh regimen (discussed later) [128]. A systematic review of clofazimine use in DR-TB suggested that it should be considered as an additional drug in DR-TB treatment [129]. Although few children have been described as treated for TB using clofazimine, there is good experience of using the drug in children with leprosy. Apart from reversible skin discoloration and gastrointestinal disturbance, it appears to be well-tolerated [130].

Two new drugs have been licenced and given conditional approval by WHO: bedaquiline and delamanid. Bedaquiline is a diarylquinoline that acts by inhibiting intracellular ATP synthase. It has a very long half-life and is effective against actively replicating as well as dormant bacilli. In clinical trials it has been shown to reduce the time to culture conversion in adults with pulmonary TB, as well as increasing the proportion that do culture-convert. [131] Although it has not been licenced for use in children, bioequivalence studies of two pediatric formulations (granules and water-dispersible tablets) have been conducted [132] and pharmacokinetic and safety studies are planned. The CDC advises that on a case-by-case basis bedaquiline might be considered in children when ‘an effective treatment regimen cannot otherwise be provided’ [133]. Delamanid is a nitroimidazole (like metronidazole) and acts predominantly on mycolic acid synthesis to stop cell wall production. It has been shown to increase culture conversion and also improve outcome in adult studies [134, 135]. Pediatric formulations have been developed and pharmacokinetic and safety studies are underway in children [136]. A single case report describes the use of delamanid in a 12-year-old boy who was failing treatment and was infected with a highly resistant organism [137]. The Sentinel Project of Pediatric Drug-Resistant Tuberculosis has produced clinical guidance to assist in the use of these new agents [138]. They suggest that both drugs could be considered in children older than 12 years and, in certain circumstances, in children younger than this. It is also suggested that consideration be given to using delamanid in place of the injectable drug in pediatric regimens; this would need careful follow-up and documentation of efficacy and safety.

### Are there any new regimens to treat children for drug-resistant TB?

In 2010, a seminal article was published describing an observational study conducted in Bangladesh [128]. Sequential cohorts of patients (mainly but not all adults) with MDR-TB were given different treatment regimens, each differing from the previous by the substitution or addition of one drug. The final cohort were given a 9-month regimen, consisting of kanamycin, clofazimine, gatifloxacin, ethambutol, high-dose isoniazid, pyrazinamide and prothionamide for 4 months, followed by gatifloxacin, ethambutol, pyrazinamide and clofazimine for 5 months. Of these patients, 88% had a favourable outcome (cured or treatment completed), compared to poorer outcomes for the five previous cohorts who had been given longer regimens (typically 15 months) with drugs including an earlier generation fluoroquinolone (ofloxacin) and without clofazimine. This study has generated much interest and has led to a number of trials and observational cohorts which seek to further evaluate this 9–12 month regimen [139, 140]. The STREAM trial is a randomised, non-inferiority trial that compares a similar 9-month regimen to the standard WHO-recommended regimen. It should complete by the end of 2016 [141]. Although all of the individual drugs with the ‘Bangladesh regimen’ are available for children in some form and are used either to treat TB already or are used for other indications, children have not been included in STREAM. The 2016 WHO guidance has suggested that children could be considered for treatment with the 9–12 month regimen under the same conditions as adults, namely when confirmed or suspected of having MDR-TB and where resistance to the fluoroquinolones is not suspected. A single case report describes the treatment of an adolescent treated with this regimen [142].

## Conclusions

There is currently unprecedented interest in pediatric TB with new drugs, new regimens and new approaches to the treatment of infection and disease for both DR- and DS-TB. We have a better understanding of the burden of childhood TB and better diagnostic tests. However, only a third of the children that develop TB are diagnosed, treated and notified. Children are still dying of this disease and TBM results in significant mortality and morbidity even if treated. Treatments for both TB infection and disease are long and the evidence base for the treatment of DR-TB is poor. We still have a long way to go and pediatric TB research still lags a long way behind research into adult TB.

New, shorter regimens are still required for the treatment of both infection and disease and for both DS- and DR-TB. Less toxic regimens are needed for the treatment of DR-TB disease and a better evidence base is needed for the treatment of DR-TB infection. Child-friendly formulations for all TB drugs are needed and our understanding of the pharmacokinetics of the second-line drugs needs further work. Although we have come a long way, there is still a long way to go.

## Abbreviations

CDC: Centers for Disease Control and Prevention
CSF: Cerebrospinal fluid
DR: Drug-resistant
DS: Drug-susceptible
DST: Drug susceptibility test
FDC: Fixed dose combination
HIV: Human immunodeficiency virus
IGRA: Interferon-gamma release assay
LTBI: Latent tuberculosis infection
MDR: Multidrug-resistant
PAS: Para-aminosalicylic acid
PTA: Pure tone audiometry
TB: Tuberculosis
TBM: Tuberculous meningitis
TSH: Thyroid stimulating hormone
TST: Tuberculin skin test
WHO: World Health Organization
XDR: Extensively drug-resistant
